# A novel synthetic trivalent single chain variable fragment (tri-scFv) construction platform based on the SpyTag/SpyCatcher protein ligase system

**DOI:** 10.1186/s12896-018-0466-6

**Published:** 2018-09-10

**Authors:** Md. Kausar Alam, Michelle Brabant, Raja Solomon Viswas, Kris Barreto, Humphrey Fonge, C. Ronald Geyer

**Affiliations:** 1 Department of Pathology and Laboratory Medicine, College of Medicine, University of Saskatchewan, Room 2841, Royal University Hospital, 103 Hospital Drive, Saskatoon, S7N 0W8 Canada; 2 Department of Biochemistry, College of Medicine, University of Saskatchewan, Saskatoon, SK S7N 5E5 Canada; 3 Medical Imaging, University of Saskatchewan, Saskatoon, SK S7N 5E5 Canada

**Keywords:** SpyTag/SpyCatcher, Multimerization, scFv, Tri-scFv, Avidity, HER3

## Abstract

**Background:**

Advances in antibody engineering provide strategies to construct recombinant antibody-like molecules with modified pharmacokinetic properties. Multermerization is one strategy that has been used to produce antibody-like molecules with two or more antigen binding sites. Multimerization enhances the functional affinity (avidity) and can be used to optimize size and pharmacokinetic properties. Most multimerization strategies involve genetically fusing or non-covalently linking antibody fragments using oligomerization domains. Recent studies have defined guidelines for producing antibody-like molecules with optimal tumor targeting properties, which require intermediates size (70–120 kDa) and bi- or tri-valency.

**Results:**

We described a highly modular antibody-engineering platform for rapidly constructing synthetic, trivalent single chain variable fragments (Tri-scFv) using the SpyCatcher/SpyTag protein ligase system. We used this platform to construct an anti-human epidermal growth factor receptor 3 (HER3) Tri-scFv. We generated the anti-HER3 Tri-scFv by genetically fusing a SpyCatcher to the C-terminus of an anti-HER3 scFv and ligating it to a synthetic Tri-SpyTag peptide. The anti-HER3 Tri-scFv bound recombinant HER3 with an apparent K_D_ of 2.67 nM, which is approximately 12 times lower than the K_D_ of monomeric anti-HER3 scFv (31.2 nM). Anti-HER3 Tri-scFv also bound endogenous cell surface expressed HER3 stronger than the monomer anti-HER3 scFv.

**Conclusion:**

We used the SpyTag/SpyCatcher protein ligase system to ligate anti-HER3 scFv fused to a SpyCatcher at its C-termini to a Tri-SpyTag to construct Tr-scFv. This system allowed the construction of a Tri-scFv with all the scFv antigen-binding sites pointed outwards. The anti-HER3 Tri-scFv bound recombinant and endogenously expressed HER3 with higher functional affinity (avidity) than the monomeric anti-HER3 scFv. The Tri-scFv had the size, valency, and functional affinity that are desired for therapeutic and imaging applications. Use of the SpyTag/SpyCatcher protein ligase system allows Tri-scFvs to be rapidly constructed in a simple, modular manner, which can be easily applied to scFvs or other antibody fragments targeting other antigens.

## Background

Advances in recombinant antibody technology make it possible to produce a variety of smaller, monovalent antibody fragments, such as single-chain variable fragments (scFvs) and antigen binding fragments (Fabs) [1, 2], which can be cost effectively produced at high levels in bacterial expression systems. These smaller monovalent fragments have desirable pharmacokinetic properties that enable faster tumor penetration, which is desirable for imaging and therapy [3, 4]. However, monovalent fragments have decreased valency and functional affinity compared to IgGs and they lack an Fc domain, resulting in a shorter half-life and less tumor accumulation [2–4].

Imaging probes based on IgGs often show low tumor to background signaling ratios, slow clearance from blood, and slow diffusion into the tumor [2, 5–7]. The Fc domain on an IgG results in long circulation times due to its interaction with neonatal Fc receptor (FcRn) on endothelial cells and pH-dependent recycling of IgGs back to the circulation [8, 9], allowing them more time to accumulate at high levels in tumors [9, 10]. The higher background signalling of antibodies and slower tumor accumulation is driving the development of antibody fragments with better pharmacokinetics for therapy and imaging. Smaller scFvs are cleared rapidly from the circulation and have higher tumor penetration due to small size, but exhibit poor tumor retention because of their monovalent binding and lower apparent affinity [11, 12]. Thus, new polyvalent antibody fragments are needed that possess intermediate circulation time and tumor penetration while having high functional affinity. Polyvalent antibody fragments with a size of 80–120 kDa represent a balance between tumor penetration, target affinity, and blood clearance [13].

Computational studies comparing tumor uptake of proteins showed that tumor targeting agents around 25 kDa have the lowest tumor uptake, whereas smaller and larger fragments have higher tumor uptake [14]. This model takes into account the difference in pharmacokinetics of different sized proteins. Smaller proteins rapidly accumulate in the tumor, but require high target affinity to be retained since they are more rapidly cleared from the blood. In contrast larger proteins require longer times to accumulate in tumors at high levels and do not require as high affinity as smaller proteins. Cuesta et al., proposed a “tumor target” zone describing features of antibody-like molecules that are required for optimal tumor targeting [13]. Key features of their model include multivalency and fragment size above the renal cutoff of 65 kDa and below 120 kDa to allow good tumor penetration. Antibody-like molecules that have these properties such as bivalent diabodies and minibodies [15] and trivalent trimerbodies [16, 17] have suitable biodistribution properties with good tumor uptake and high tumor to blood ratios [14, 18].

Advanced antibody engineering enables the creation of bivalent and trivalent antibody fragments that meet the tumor target zone requirements [4, 13, 19]. A number of strategies have been developed to construct trivalent antibodies with unique binding geometries, highlighting the potential to take advantage of the enhanced avidity possible with trivalent antibody fragments. Currently, strategies have been limited to genetically fused antibody fragments to generate Fab-scFv_2_ tribodies [20] or by putting a polypeptide linker of different length to generate scFv-based tribodies [21–24]. Oligomerization domains have also been used to generate scFv and VHH based tribodies [16, 25–27].

Here, we describe a modular strategy to assemble Tri-scFvs post-translationally from scFv-SpyCatcher and Tri-SpyTag parts using the SpyTag/Catcher protein ligase system. This strategy allows smaller scFv-SpyCatcher fusion proteins to be easily and cost effectively produced in bacteria, which can be covalently ligated to a Tri-SpyTag. We used this system to produce an anti-HER3 Tri-scFv and showed that it has higher functional affinity (avidity) towards recombinant HER3 and endogenous cell surface HER3.

## Results

### Construction of anti-HER3 tri-scFv

**Fig. 1:**
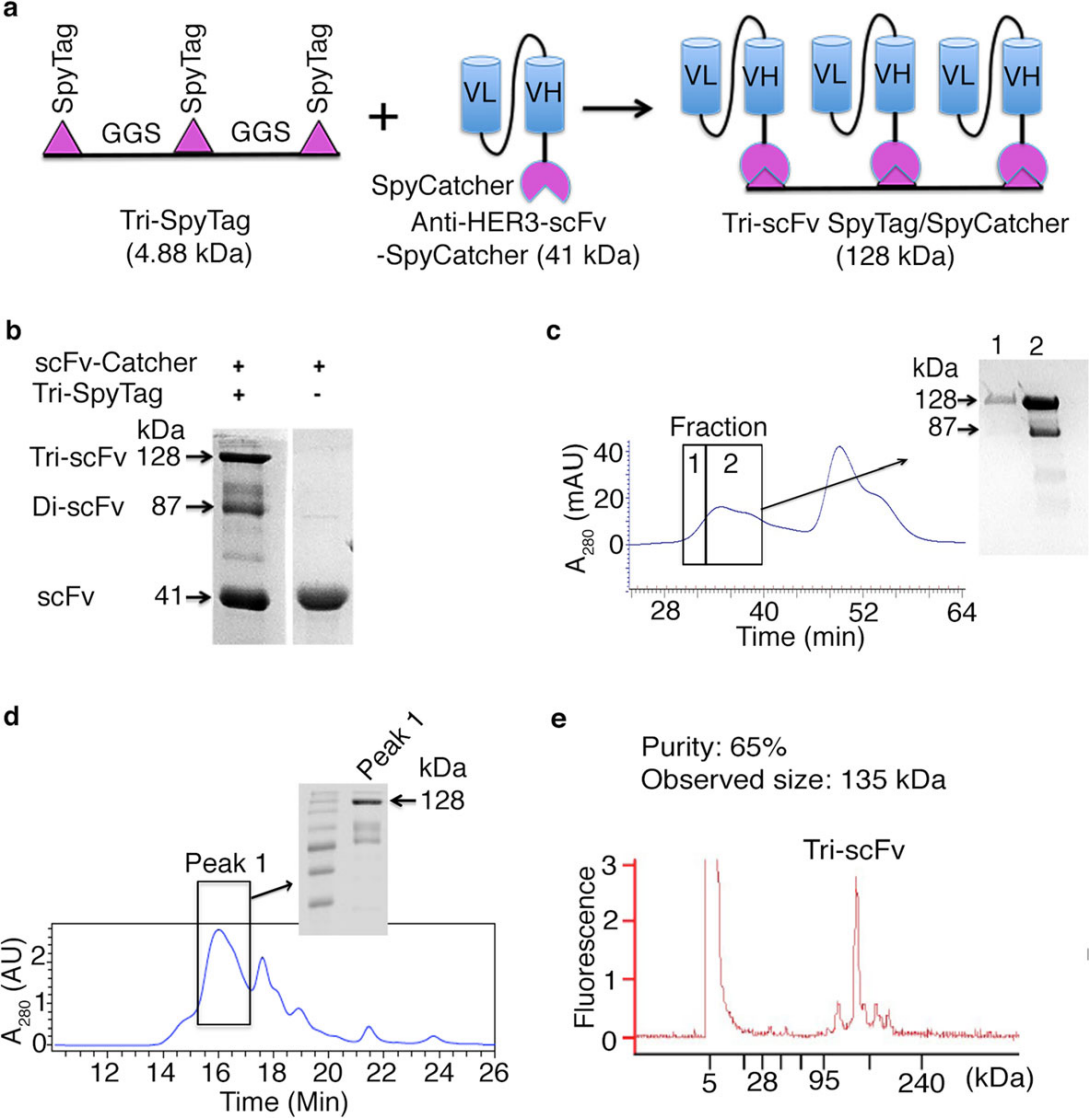
Construction and Purification of SpyTag/SpyCatcher based anti-HER3 Tri-scFv. **a** Schematic of the SpyTag/SpyCatcher based anti-HER3 Tri-scFv ligation reaction. SpyCatcher was fused to the C-terminus of anti-HER3 scFv (VL-(G_4_S)3-VH-GGS-SpyCatcher). Anti-HER3 Tri-scFv was generated by mixing monomeric scFv-SpyCatcher and a synthetic Tri-SpyTag peptide together at 6:1 M ratio. VL: variable light domain, VH: variable heavy domain. **b** Reducing SDS-PAGE analysis of TriSpyTag peptide and scFv-SpyCatcher ligation products. scFv-SpyCatcher and Tri-SpyTag peptide have molecular weights of 41 kDa and 4.88 kDa, respectively. After successful ligation, trivalent scFv (Tri-scFv) and divalent scFv (Di-scFv) had a molecular weight of 128 kDa and 87 kDa, respectively. **c** Size-exclusion chromatogram (SEC) of Tri-scFv ligation reaction. Inset shows SDS-PAGE analysis of two fractions eluted from 30 to 40 mins. **d** Further separation of Tri-scFv using SEC-HPLC. Inset shows SDS-PAGE analysis of fraction eluted from 15 to 17 mins. **e** Bioanalyzer electropherograms of SEC-HPLC separated Tri-scFv under non-reducing conditions. The observed molecular weight (MW) of Tri-scFv is 135 kDa

We constructed the anti-HER3 Tri-scFv using the SpyTag/SpyCatcher protein ligase system [28–30] (Fig. 1a). We used a previously reported anti-HER3 scFv [30] and genetically fused a His-Tag-SpyCatcher to it to generate the anti-HER3 scFv-SpyCatcher construct as described previously [30]. The SpyCatcher was fused to the C-terminus of the anti-HER3 scFv to avoid interacting with complementarity determining regions (CDRs) in the V_L_ and V_H_ domains of the scFv. We expressed the anti-HER3 scFv-SpyCatcher in *E. coli* and used protein L chromatography to purify the anti-HER3 scFv-SpyCatcher [26]. We constructed a synthetic peptide Tri-SpyTag containing three SpyTags connected by a Gly-Gly-Ser linker (Fig. 1a).

To construct the anti-HER3 Tri-scFv (Fig. 1a), we reacted the anti-HER3 scFv-SpyCatcher (41 kDa) with a synthetic Tri-SpyTag peptide (4.88 kDa) at a 6:1 M ratio under conditions described previously [28, 30]. We used excess anti-HER3 scFv-SpyCatcher to maximize conversion of anti-HER3 Tri-scFv. We used SDS-PAGE under reducing conditions to analyze the anti-HER3 scFv-SpyCatcher and synthetic Tri-SpyTag peptide ligation reaction. Tri-scFv and SpyTag with two anti-HER3 scFv-SpyCatchers (Di-scFv) were observed at a ratio of 6:4 in the SDS-PAGE at 128 kDa and 87 kDa, respectively (Fig. 1b).

### Anti-HER3 tri-scFv purification

To remove excess unreacted scFv-SpyCatcher and Di-scFv and scFv, we used size exclusion chromatography (SEC). SEC removed most of the scFv from Di-scFv and Tri-scFv (Fig. 1c). However, the yield of Tri-scFv after SEC was low and a significant amount of SEC fractions contained a mixture of both Tri-scFv and Di-scFv. To further separate Tri-scFv from Di-scFv, we performed higher resolution SEC-HPLC (Fig. 1d). We analyzed the purity and size of SEC HPLC separated Tri-scFv using the Agilent 2100 Bioanalyzer. The final purified Tri-scFv product consisted of one major peak with a molecular mass of 135 kDa (Fig. 1e). The final reaction yield of Tri-scFv was ~ 20%.

### Binding of anti-HER3 tri-scFv to recombinant HER3

**Fig. 2:**
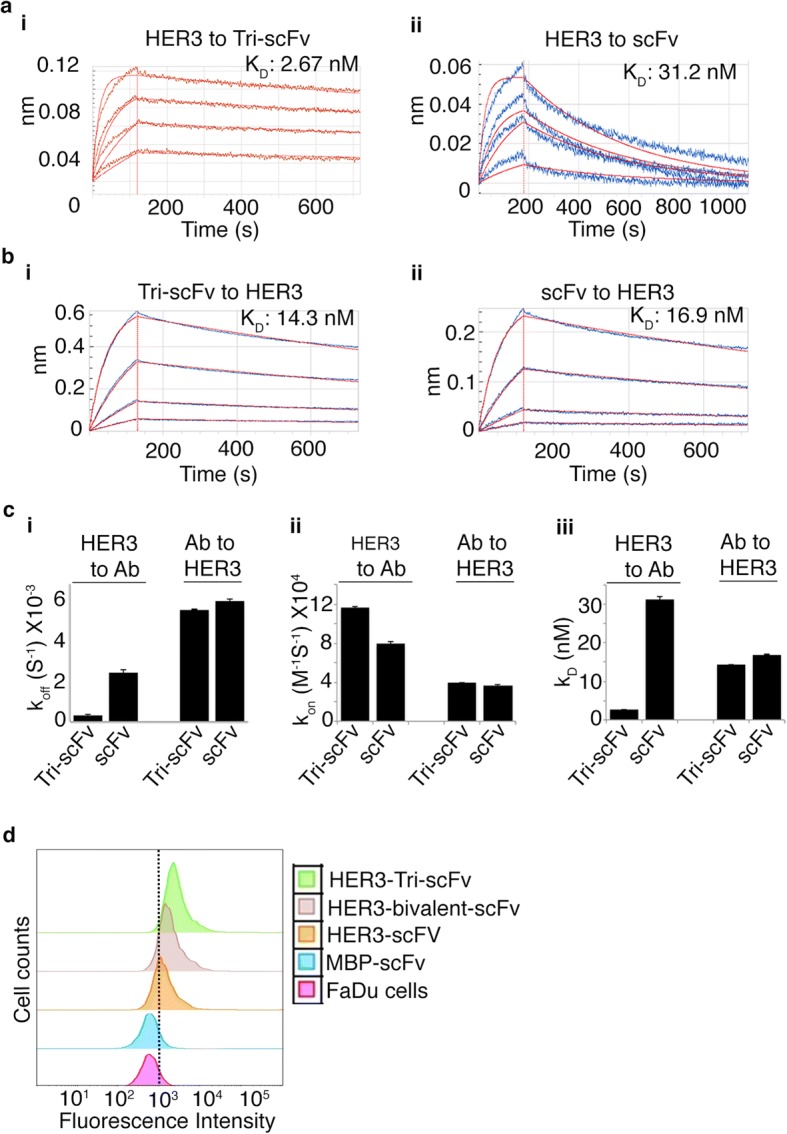
Analysis of the HER3 binding Tri-scFv and monomeric scFv-SpyCatcher to recombinant HER3 and a HER3-postive cell line. Biolayer interferometry kinetic analysis of anti-HER3 Tri-scFv and anti-HER3 scFv-SpyCatcher against recombinant HER3. **a** HER3 sensors were generated by covalently immobilizing HER3 on AR2G sensors. HER3 sensors were placed in wells containing concentrations of Anti-HER3 Tri-scFv (i) and anti-HER3 scFv-SpyCatcher (ii), ranging from 20 nM to 500 nM. **b** anti-HER3 Tri-scFv and anti-HER3 scFv-SpyCatcher sensors were generated by covalently immobilizing Anti-HER3 Tri-scFv (i) and anti-HER3 scFv-SpyCatcher (ii) on AR2G sensors. Tri-scFv and monomeric scFv loaded sensors were then placed in wells containing concentrations of HER3, ranging from 20 nM to 500 nM. **c** Comparison of k_off_ (i), k_on_ (ii), and K_D_ (iii) between anti-HER3 Tri-scFv and scFv-SpyCatcher with HER3 using HER3 and scFv sensors. Data analysis and fitting were performed using ForteBio’s Data analysis software version 8.1. Error bars represent the percentage of fitting error as calculated by FortéBio Data Analysis software. **d** Flow cytometry analysis of binding of anti-HER3 Tri-scFv and scFv-SpyCatcher to the HER3-positive FaDu cell line. A fixed concentration (0.5 μM) of IRDye680RD-labeled anti-HER3 Tri-scFv-IRDye680 (HER3 Tri-scFv) and anti-HER3 scFv-SpyCatcher-IRDye680 (HER3-scFv) were incubated with 1 × 10^5^ FaDu cells. Anti-Maltose Binding Protein scFv-SpyCatcher-680 (MBP scFv-SpyCatcher) was used a negative control. The mean fluorescent intensity (MFI) was 592 for FaDu cells, 696 for the MBP-scFv, 3834 for the scFv, 4636 for the bivalent-scFv, and 6919 for the Tri-scFv. The Labeling efficiency of the Tri-scFv-IRDye680, bivalent-scFv-IRDye680, scFv-SpyCatcher-IRDye680 and MBP-scFv-IRDye680 was 2.1, 1.7, 1.6, and 1.5 dyes per antibody fragment, respectively

We analyzed the binding of the anti-HER3 Tri-scFv to recombinant HER3 using bio-layer interferometry. We covalently immobilized recombinant HER3 onto the sensor tip and placed the HER3-modified tip in a range of anti-HER3 Tri-scFv and anti-HER3 scFv-SpyCatcher analyte concentrations. K_D_s of the anti-HER3 Tri-scFv and the anti-HER3 scFv-SpyCatcher were 2.67 nM and 31.2 nM, respectively (Fig. 2a and c). The lower apparent K_D_ of the anti-HER3 Tri-scFv was due to a lower k_off_ (3.08E-04 ± 4.80E-06 s^− 1^) versus the scFv-SpyCatcher (2.47E-03 ± 1.57E-05 s^− 1^) (Fig. 2a and c). When the anti-HER3 Tri-scFv and scFv-SpyCatcher were covalently immobilized on the sensor and immersed in a range of recombinant HER3 concentrations, the enhanced functional affinity of the anti-HER3 Tri-scFv was not observed. The K_D_s of Tri-scFv and scFv-SpyCatcher were 14.3 nM and 16.9 nM, respectively (Fig. 2b and c). Since the HER3 analyte was monovalent, we would not expect to observe any avidity against biosensor immobilized scFv or tri-scFv.

The results do not confirm that the avidity is due to engagement of all three scFvs to HER3 at the same time. Previous studies showed that an anti-HER3 IgG and a bivalent scFv generated with the same anti-HER3 scFv using the spyTag/SpyCatcher systems [30] have a apparent K_D_s of 6 nM and 7.01 nM, respectively, which were between the Tri-scFv (2.67 nM) and the scFv (31.2 nM). The major difference between the scFv, bivalent-scFv, IgG, and Tri-scFv was lower K_off_s, which were 2.47E-03 s^− 1^, 6.90E-04 s^− 1^, 6.01E-04 s^− 1^, and 3.08E-04 s^− 1^, respectively. Although anti-HER3 scFvs in the polyvalent fragments may not be optimally arranged to allow simultaneous engagement, the addition of scFvs to antibody fragments results in lower k_off_s.

### Binding of anti-HER3 tri-scFv to endogenous cell surface HER3

We used flow cytometry to compare the binding of anti-HER3 Tri-scFv, bivalent-scFv [30], and scFv-SpyCatcher to the HER3-positive FaDu cell line (Fig. 2d). We labeled anti-HER3 Tri-scFv, bivalent-scFV, and scFv-SpyCatcher with IRDye680RD NHS ester. Previously, we showed that labeling of an anti-HER3 antibody fragments consisting of the same light and heavy variable domains with IRDye800CW-NHS ester did not inhibit binding to the FaDu cells [In Press]. FaDu cells (1 × 10^5^) were labeled with anti-HER3 Tri-scFv, bivalent-scFv, and monomeric scFv-SpyCatcher at a fixed concentration (0.5 μM). An IRDye680RD labeled anti-Maltose Binding Protein scFv-SpyCatcher (anti-MBP scFv-SpyCatcher) was used as a negative control for flow cytometry analyses. The anti-HER3 Tri-scFv labeled FaDu cells with a mean fluorescence intensity (MFI) of 6919, followed by the bivalent-scFv (MFI –4636) and the scFv (MFI –3834), which was consistent with the trend observed for recombinant HER3 binding.

## Discussion

In this study, we used the SpyTag/SpyCatcher protein ligase system [28–30] to construct an anti-HER3 Tri-scFv with increased size and valency relative to the anti-HER3 scFv. The spyTag/SpyCatcher ligase is active under a variety of reaction conditions and forms an irreversible isopeptide bond [28]. The anti-HER3 Tri-scFv showed a lower K_D_ due to a slower k_off_ against recombinant HER3 than the anti-HER3 scFv, consistent with enhanced avidity of the anti-HER3 Tri-scFv. The MFI of anti-HER3 Tri-scFv stained cells expressing endogenous cell surface HER3 was 2.4-fold higher than anti-HER3 scFv at the 0.5 μM concentration tested.

Antibody fragments with trivalency have been identified as ideal reagents for rapid tumor retention [13, 15–18]. Current methods to generate trivalent antibody fragments use flexible polypeptide linkers [21–24], linear gene fusions [20], or self-associating peptides [16, 25–27]. Genetically fusing fragments limits the orientation of fragments and requires the expression and correct folding of larger antibody fragments. Flexible linkers that drive formation of trivalent antibody fragment or the use of oligomerization domains to non-covalently associate fragments result in antibody fragments that are heterogeneous in nature [21–24]. In comparison, the SpyTag/SpyCatcher protein ligase system allows greater flexibility in joining antibody fragments. The SpyCatcher domain can be used as a N- or C-termini fusion and the SpyTag peptide can be inserted at the N- or C-termini or in the middle of the antibody fragment [28–31]. In the Tri-scFv, the SpyCatcher domain was fused to the C-terminus to the scFv so the scFv could be post-translationally conjugated to the SpyTag at a site distant from the CDRs. These types of linkages are not possible by genetically fusing the scFvs together.

Further optimization of the Tri-scFv reaction is required as the yield is relatively low (~ 20%) and requires high resolution high SEC-HPLC to remove Di-scFv product. Optimization of the space between the SpyTags in the Tri-SpyTag-peptide may remove possible steric hindrance between scFvs and improve yields. More space between the SpyTags would also provide more flexibility to the Tri-scFv, resulting in better binding by making it easier for scFvs to simultaneously engage HER receptors.

## Conclusion

We demonstrated a modular system to post-translationally assemble synthetic scFvs to generate a trivalent Tri-scFv. The system uses pre-constructed antibody fragments genetically fused to the SpyCatcher domain and a synthetic peptide encoding three sequential SpyTag moieties. The system is universal in that in can be used with a variety of antibody fragments to antibody-like molecules with high valency. The system is cost-effective as many small fragments can be produced in bacteria and SpyTags can be constructed using solid-phase peptides synthesis.

## Methods

### Tri-SpyTag

Tri-SpyTag was purchased from Genscript (GenScript, New Jersey, USA). TriSpyTag sequence is: AHIVMVDAYKPTKGGSAHIVMVDAYKPTKGGSAHIVMVDAYKPTKC.

### Anti-HER3 mono-scFv-SpyCatcher expression and purification

The expression plasmid, pCW-Anti-HER3-scFv-Catcher-His_6_ [30] was generated by inserting SpyCatcher-His_6_ at the C-terminus of V_H_ of scFv as described previously [30]. Anti-HER3 scFv-SpyCatcher expression plasmid was transformed into BL21 (DE3) competent *E. coli* cells (NEB) and plated on 2YT media containing antibiotic carbenicillin. Single *E. coli* colonies were cultured in overnight expression Instant TB media (Novagen) for 20–24 h. Cells were pelleted and suspended in Protein L Binding buffer (Sodium Phosphate 20 mM, 0.15 M NaCl, pH 8.0). Cells were disrupted at 35 Kpsi using the Cell disruptor (Constant System LTD. USA). Cell debris was removed by centrifuging at 12,000 RPM for 20 min. Supernatant was collected and filtered through a 0.45-μm membrane filter (Minisart, Sartorius stedim). Anti-HER3 mono-scFv-SpyCatcher was purified on a GE Healthcare AKTA FPLC system using a HiTrap Protein L column (GE Heathcare). Anti-HER3 mono-scFv-SpyCatcher was eluted from the Protein L column using IgG elution buffer (Fisher Scientific) and neutralized with neutralization buffer (1 M Tris-HCl pH 9.0). Purified Anti-HER3 mono-scFv-SpyCatcher was dialyzed overnight in PBS and concentrated using 10 K MWCO filter (Millipore). Anti-HER3 mono-scFv-SpyCatcher was filter sterilized and stored at − 20 °C. Anti-HER3 mono-scFv-SpyCatcher was quantified using the Bicinchonic acid assay (Pierce™ BCA Protein Assay Kit, Thermo Scientific) following manufacturer’s instructions.

### Generation of tri-scFv

Anti-HER3-TriscFv was synthesized by reacting anti-HER3 scFv-SpyCatcher-His_6_ (60 μM) with the Tri-SpyTag (10 μM) for 4 h at room temperature in phosphate-citrate buffer pH 7, as describe previously [28–30].

### SDS-page

Purified antibody fragments were separated under reducing conditions on 12% SDS PAGE. Proteins were stained with Coomassie blue and visualized using GelDoc XR^+^ system (BioRad). Ratios of Tri-scFv and Di-scFv were calculated using Image J Browser (ImageJ 1.49 V).

### Size exclusion chromatography (SEC)

Conjugated anti-HER3 Tri-scFv reaction was separated using the HiLoad™ 16/600 Superdex™ 200 pg (GE Healthcare). The gel filtration column was equilibrated with PBS pH 7.4. Anti-HER3 Tri-scFv (5 ml) was subjected to size-exclusion chromatography at flow-rates of 1 mL/min on the AKTA Prime FPLC system (GE Healthcare).

### Size exclusion HPLC

SEC separated peak of anti-HER3 Tri-scFv was separated using Superdex™ 200 increase (GE Healthcare) at ambient temperature by using HPLC Bio-separation system (Waters). The gel filtration column was equilibrated with PBS pH 7.4. The separation was performed at 0.6 mL/min flow rate for 30 min by using a mobile phase containing PBS, pH 7.0. The absorbance at 220 and 280 nm of the eluate was monitored during the LC run.

### Agilent 2100 bioanalyzer

The molecular weight (MW) and purity of the anti-HER3 Tri-scFv was determined using the Agilent 2100 Bioanalyzer using Agilent High Sensitivity Protein 250 Kit (cat # 5067–1575) according to manufacture’s conditions. Briefly, Anti-HER3 Tri-scFv (0.5 mg/mL) was labeled with a fluorescent dye at non-reducing condition as described in the High Sensitivity Protein 250 kit guide. The chip preparation was performed according to the manual provided with the kit. Samples were then separated in the chip and detected using a fluorescence detector. The molecular weight and peak areas were calculated using Agilent 2100 Expert software.

### Biolayer interferometry

On (k_on_) and off (k_off_) rates for the anti-HER3 mono-scFv-SpyCatcher and the anti-HER3 Tri-scFv with recombinant HER3 was measured using biolayer interferometry with the ForteBio Octet^Red^ 384 and AR2G sensors, according to manufacturer’s conditions. Briefly, biolayer interferometry procedures were performed at 30 °C with shaking at 1000 rpm. For HER3 to antibody, AR2G sensors were loaded with recombinant HER3 (Sino Biological) by incubating AR2G sensors in 50 μL of HER3 (500 nM) for 300 s. HER3 loaded sensors were washed in PBS for 300 s and then placed in wells containing a range of anti-HER3 mono-scFv-SpyCatcher and anti-HER3 Tri-scFv concentrations, ranging from 20 nM to 500 nM. For biolayer interferometry in the reverse orientation, AR2G sensors were loaded with anti-HER3 monoscFv-SpyCatcher and anti-HER3 Tri-scFv by incubating AR2G sensors in 50 μL of antibody fragments (500 nM) for 300 s. Antibody fragment loaded sensors were washed in PBS for 300 s and then placed in wells containing a range of recombinant HER3 concentrations, ranging from 20 nM to 500 nM. Rates of association were monitored for 5 min and rates of dissociation were monitored for 10 min. Data analysis and fitting were performed using ForteBio’s Data analysis software version 8.1. We used a 1:1 binding model to fit the data, which is commonly used for kinetics analysis of monoclonal antibodies (IgG) to antigen [32]. Previous instances have shown that K_D_s were similar for both 1:1 and 2:1 models, with a worse fit but more accurate data obtained with the 1:1 model https://www.fortebio.com/interactions/Fall_2014/index2.html.

### Tissue culture

The HER3 positive human head and neck squamous cell carcinoma cell line, FaDu cell line, was obtained from ATCC (Rockville, MD, USA) [33]. FaDu cells were propagated by serial passage in MEM/EBSS medium (HyClone Laboratories, Logan UT), supplemented with 10% fetal bovine serum (FBS) at 37 °C in a humidified atmosphere of 5% CO_2_.

### Labeling of anti-HER3 scFv-SpyCatcher and anti-HER3 tri-scFv with IRDye680RD NHS ester

IRDye680RD NHS ester (LI-COR Biosciences Co., Lincoln, NE) was used to label anti-HER3 scFv-SpyCatcher and anti-HER3 Tri-scFv. A total of 0.5 mg of Anti-HER3 scFv-SpyCatcher and Anti-HER3 Tri-scFv in 0.5 mL phosphate-buffered saline (PBS, pH 7.4) was labeled with 3-fold molar excess of IRDye 680RD–NHS ester by mixing and rotating for 2 h at room temperature protected from light followed by rotating overnight at 4 °C. The reaction was quenched with molar excess of pure Tris base. The dye to protein ratio was determined by measuring the labeled protein absorbance in PBS at 280 nm and 774 nm as per the manufacturer’s recommendation [34]. The labeled anti-HER3 mono-scFv-SpyCatcher and anti-HER3 Tri-scFv were store at − 20 °C for further use.

### Flow cytometry

The binding of anti-HER3 mono-scFv-SpyCatcher and anti-HER3 Tri-scFv to Fadu cells was analyzed by flow cytometry. 1 × 10^5^ cells/tube were incubated with 50 μM of anti-HER3 monoscFv-SpyCatcher-IRDye680 or anti-HER3 Tri-scFv-IRDye680 at room temperature for 60 mins. Cells were washed three times with PBS, pH 7.4. Flow cytometry was performed using a Beckman Coulter Gallios flow cytometer system with 680 nm excitation and 694 nm emission wavelengths. Data obtained was analyzed using FlowJo (Tree Star).

## Abbreviations

AR2G: Amine reactive second-generation
Di-ScFv: Divalent Single chain variable fragments
EC: Effective concentration
HER3: Epidermal growth factor receptor 3
HPLC: High Performance Liquid Chromatography
Mono-scFv: Monovalent Single chain variable fragments
scFv: Single chain variable fragments
SDS-PAGE: sodium dodecyl sulfate polyacrylamide gel electrophoresis
SEC: Size exclusion chromatography
Tri-scFv: Trivalent Single chain variable fragments
